# The Human Upper Respiratory Tract Epithelium Is Susceptible to Flaviviruses

**DOI:** 10.3389/fmicb.2019.00811

**Published:** 2019-04-16

**Authors:** Nathalie J. Vielle, Obdulio García-Nicolás, Blandina I. Oliveira Esteves, Melanie Brügger, Artur Summerfield, Marco P. Alves

**Affiliations:** ^1^ Institute of Virology and Immunology, Bern, Switzerland; ^2^ Department of Infectious Diseases and Pathobiology, Vetsuisse Faculty, University of Bern, Bern, Switzerland; ^3^ Graduate School of Cellular and Biomedical Sciences, University of Bern, Bern, Switzerland

**Keywords:** flavivirus, non-vector borne transmission, nasal epithelial cells, respiratory epithelium, upper respiratory tract, air-liquid interface cultures

## Abstract

Flaviviruses replicate in a wide variety of species and have a broad cellular tropism. They are isolated from various body fluids, and Zika virus (ZIKV), Japanese encephalitis virus (JEV), and West Nile virus (WNV) RNAs have been detected in nasopharyngeal swabs. Consequently, we evaluated the cellular tropism and host responses upon ZIKV, JEV, WNV, and Usutu virus (USUV) infection using a relevant model of the human upper respiratory tract epithelium based on primary human nasal epithelial cells (NECs) cultured at the air-liquid interface. NECs were susceptible to all the viruses tested, and confocal analysis showed evidence of infection of ciliated and non-ciliated cells. Each flavivirus productively infected NECs, leading to apical and basolateral live virus shedding with particularly high basal release for JEV and WNV. As demonstrated by a paracellular permeability assay, the integrity of the epithelium was not affected by flavivirus infection, suggesting an active release of live virus through the basolateral surface. Also, we detected a significant secretion of interferon type III and the pro-inflammatory cytokine IP-10/CXCL10 upon infection with JEV. Taken together, our data suggest that the human upper respiratory tract epithelium is a target for flaviviruses and could potentially play a role in the spread of infection to other body compartments through basolateral virus release. Undoubtedly, further work is required to evaluate the risks and define the adapted measures to protect individuals exposed to flavivirus-contaminated body fluids.

## Introduction

Flaviviruses constitute a relevant public health concern in endemic and unaffected regions of the world. These viruses are closely genetically related and possess a single-stranded positive RNA genome encoding for three structural proteins (C, prM, and E protein) and seven non-structural proteins. The Flavivirus genus includes more than 70 different species, and among these, about half are transmitted to vertebrates by mosquitoes, almost a third is transmitted *via* ticks, and for the rest, the vector is unknown (Mackenzie and Williams, 2009; Simmonds et al., 2017). Additionally, some flaviviruses, the insect-specific flaviviruses, are restricted to non-vertebrate hosts (Gravina et al., 2019). Flaviviruses are maintained in sylvatic, enzootic cycles in which the virus crosses between an animal population and an arthropod vector (Gould et al., 2003; Simmonds et al., 2017). Many flaviviruses are etiologic agents of zoonotic diseases with a broad range of clinical manifestations from febrile illness to encephalitis or hemorrhagic fever, which can be lethal in some cases (Duffy et al., 2009; Weissenbock et al., 2010; Dumre et al., 2013; Simmonds et al., 2017). While most mosquito-borne flavivirus infections in humans occur *via* a vector of the *Aedes* or *Culex* genus, alternative routes may play an important role in their transmission. For instance, transmission of Zika virus (ZIKV) from mother to child can occur transplacentally or, for ZIKV and West Nile virus (WNV), perinatal transmission in some cases *via* breastfeeding has also been documented (O’Leary et al., 2006; Hinckley et al., 2007; Blohm et al., 2018). Transmission of ZIKV, WNV, and Dengue virus (DENV) *via* blood product transfusion can also occur, but so far, ZIKV is the only flavivirus for which sexual transmission is proven in humans (Pealer et al., 2003; Sakkas et al., 2018). However, a recent case report presents evidence of a probable sexual transmission of DENV (Lee and Lee, 2018). Close contact transmission of ZIKV has been speculated in a reported case of transmission without sexual contact, and in the case of WNV and Wesselsbron virus (WESSV), infections have been reported in a poultry farm and in a laboratory setting, also indicative of potential contact transmission (Weiss et al., 1956; Centers for Disease Control and Prevention, 2003; Swaminathan et al., 2016). Flavivirus direct transmission by close oronasal contact has been described for Japanese encephalitis virus (JEV) and ZIKV in pigs and guinea pigs, respectively (Ricklin et al., 2016a; Deng et al., 2017). In JEV-infected pigs, the peak of viral RNA was reached in oronasal swabs after the viremia phase ended; interestingly, in a JEV vaccine study, immunized animals oronasally shed JEV after 10 days post infection despite the fact that a viremia never developed (Ricklin et al., 2016a,b; Garcia-Nicolas et al., 2017). Based on recent publications showing detection of DENV and JEV RNAs in the human upper respiratory tract within the first days of fever, it could be speculated that oronasal flavivirus shedding in humans could have remained unnoticed, as patients will usually be admitted to intensive care units only at later stages of the infection (Tavakoli et al., 2007; Cheng et al., 2017; Bharucha et al., 2018). Moreover, ZIKV RNA has been detected in nasopharyngeal swabs of a patient bitten by an infected monkey (Leung et al., 2015), and sexual transmission of ZIKV through receptive oral sex has been reported (D’Ortenzio et al., 2016). Altogether, this suggests that the mucosa from the upper respiratory tract may play a role in oronasal transmission of flaviviruses in humans.

The upper respiratory epithelium is a ciliated pseudostratified columnar epithelium composed of ciliated, goblet, club, and basal cells. It has a protective function, which not only prevents the access to pathogens and foreign particulate matter but also moistens the inhaled air. Beyond its role as a physical barrier, the epithelium within the mucosae bears potent innate immune functions, allowing the identification of pathogens through pattern recognition receptors (PRRs). The outcome of PRRs activation by microbial products such as viral RNA is the activation of immune response mechanisms leading to the release of antiviral mediators. Type I (α and β) interferons (IFNs) play a central role in defense against viral pathogens and type III (λs) IFNs seem to be also important at mucosal surfaces (Iversen et al., 2010). In addition, pro-inflammatory cytokines are typically induced upon viral infection of the airway epithelium (Schogler et al., 2016b). The most robust *in vitro* model of the respiratory epithelium is based on primary airway epithelial cells and is used to study molecular and functional aspects of virus infection *in vitro* (Schogler et al., 2015; Alves et al., 2016; Schogler et al., 2016a,b). Primary nasal epithelial cells (NECs) can non-invasively be isolated from the inferior surface of the middle turbinate of nostrils with cytology brushes (Stokes et al., 2014). Progress in modeling the upper respiratory tract led to the establishment of well-differentiated cell cultures. In an air-liquid interface (ALI) culture system, airway epithelial cells grow on a porous membrane, and their basal surface is in contact with culture medium in the basolateral chamber; after reaching confluency, the removal of apical medium and the exposure of the apical side of the cells to air mimic the conditions found in the airways. Airway epithelial cells grown at the ALI are now widely used to study airway disease mechanisms or for drug discovery, as they are robust surrogates of the structure and barrier functions of native respiratory epithelium (Ong et al., 2016; Schogler et al., 2017). In this regard, with the use of porcine NECs in an ALI culture system, we recently demonstrated that JEV possesses the ability of infecting apically, resulting in both apical and basolateral virus shedding (Garcia-Nicolas et al., 2018). These data indicate that the porcine nasal mucosa could represent a gateway for JEV entry and release in pigs, which could help explain the oronasal pig-to-pig JEV transmission (Ricklin et al., 2016a; Garcia-Nicolas et al., 2018).

In this study, we exposed apically well-differentiated human NECs cultured at the ALI to the related flaviviruses ZIKV, JEV, WNV, and Usutu virus (USUV). We selected these viruses due to the recent increasing evidences of potential threat to humans (Cadar et al., 2017; Simonin et al., 2018). We show that NECs are particularly susceptible to JEV and WNV infection and to other flaviviruses included in this study. Infection with each virus led to shedding of infectious virus particles through the apical and basolateral surfaces and triggered host mechanisms at the level of inflammatory and antiviral mediators. Given the evidences of contact transmission in humans and detection of flaviviruses RNA in upper respiratory tract samples, our data suggest that the human respiratory epithelium could represent a target for the replication of flaviviruses.

## Materials and Methods

### Virus Propagation

JEV (G1; CNS769_Laos_2009, GenBank accession number KC196115.1) was kindly provided by R. Charrel Aix-Marseille Université, Marseille, France. WNV (NY99–35, GenBank accession number DQ211652.1) and USUV (SAAR-1776, GenBank accession number AY453412) were kindly provided by R. Hoop, Institute of Veterinary Bacteriology, University of Zurich, Zurich, Switzerland. The low passage clinical isolate of Asian lineage ZIKV (PRVABC59) was obtained at PHE (Public Health England). All flaviviruses were passaged on Vero cells cultured in DMEM (Gibco) supplemented with 10% FBS (Biochrom) to generate virus stocks. Flavivirus titers were determined on Vero cells (CCL-81, ATCC) and expressed as 50% tissue culture infective dose per ml (TCID_50_/ml) using the Reed and Muench method (Reed and Muench, 1938). Respiratory syncytial virus (RSV) A2 (ATCC, VR-1540, GenBank accession number KT992094.1) was propagated on HEp-2 cells (ATCC, CCL-23) cultured in DMEM (Gibco) supplemented with 5% FBS (Gibco) to generate a virus stock. Virus titers were determined on HEp-2 cells and expressed as plaque forming units (PFU) per ml (PFU/ml).

### Virus Infection of Human NECs

MucilAir™ (Epithelix Sàrl, Geneva, Switzerland) NECs isolated from independent healthy donors were used for virus infection. The cultures were maintained on 24-well transwell inserts (Corning) at ALI in MucilAir™ culture medium (Epithelix Sàrl, Geneva, Switzerland) in a humidified incubator at 37°C and 5% CO_2_. Culture medium was changed every 3 days, and the apical surface was rinsed with phosphate-buffered saline solution (PBS, Sigma-Aldrich) once a week and prior to infection in order to wash out the mucus. For apical infection, virus preparations were diluted in medium (final volume 200 μl) in order to reach a MOI of 1 TCID_50_/cell, typical MOIs used when working with human NEC ALI cultures and respiratory viruses (Lopez-Souza et al., 2009; Schneider et al., 2010; Villenave et al., 2012; Altman et al., 2018). The virus dilutions were applied apically to the NEC inserts, which contain on average 500,000 cells per insert. As a mock control, the culture supernatant of uninfected cells was used. Virus particles were allowed to adsorb for 3 h at 37°C and 5% CO_2_ before the inoculum was removed. Each insert was carefully washed three times with pre-warmed PBS and placed on a new well containing fresh MucilAir™ culture medium. For determination of viral particle release 24, 48, and 72 hours post infection (h p.i.), 200 μl PBS was added to the apical chamber and incubated for 10 min at 37°C, harvested, and stored at −70°C until further analysis. Aliquots of basolateral media were collected and stored similarly. Finally, at 72 h p.i., the inserts were collected for confocal imaging.

### RNA Extraction and Quantitative RT-PCR

RNA was extracted from basolateral medium using the QIAamp® viral RNA kit (Qiagen). Complementary DNA (cDNA) was generated with the Omniscript-RT Kit (Qiagen) using random hexamers (Invitrogen). Quantitative RT-PCR was performed using TaqMan® Fast Universal PCR Master Mix (Applied Biosystems). The analysis of the RNA expression levels of the cytokines normalized to the housekeeping gene 18S was done using the ∆ΔCt method. The primer and probe sequences used to detect IFN-β, IFN-λ1,2/3, IL-6, IL-8/CXCL8, IP-10/CXCL10, and the housekeeping gene 18S have been previously described (Schogler et al., 2015; Vielle et al., 2018).

### Cytokine Measurement

Basolateral medium aliquots of NEC harvested at 72 h p.i. were used for determining human IFN-λ1/3, IL-6, IL-8/CXCL8, and IP-10/CXCL10 using commercial enzyme-linked immunosorbent assay (ELISA) kits (R&D Systems).

### Confocal Imaging

Inserts were washed with PBS and incubated in 4% paraformaldehyde (PFA) for 15 min. In a second step, the inserts were incubated in confocal buffer (50 nM ammonium chloride, 0.1% saponin in PBS) for 30 min. Cells were incubated with anti-Zonula occludens 1 (ZO-1, clone 1A12, Thermofisher) and anti-E protein Flavivirus group antibody 4G2 (ATCC, HB-112™) for 2 h followed by the Alexa 633 coupled goat anti-rabbit IgG (Thermofisher), Alexa 488 coupled anti-IgG2a (molecular probes), and anti-β Tubulin directly coupled with Cy3 (clone TUB 2.1, Abcam) in the dark. DAPI (Sigma) was applied to the insert for 5 min and washed with PBS. Each step was performed at room temperature. The membranes were mounted on glass slides in MOWIOL® 4-88 Reagent (Sigma-Aldrich). For confocal microscopy analysis, a Nikon confocal microscope A1 (Nikon) combined with an ECLIPSE Ti inverted microscope (Nikon) and a digital imaging Nikon software (NIS-Elements AR 3.30.02) was used. All images were acquired with the 40X objective and sequential channel acquisition was performed. The images were analyzed with Imaris 8.0.2 software (Bitplane AG, Zurich, Switzerland). To avoid false-positive emissions, different settings were applied including background subtraction, threshold applications, gamma correction, and maxima.

### Titration of Virus With 50% Tissue Culture Infectious Dose Assay

A 10-fold dilution of the collected apical washings and basolateral chamber media were distributed onto Vero cells (flaviviruses) or HEp-2 cells (RSV) and maintained for 72 h at 37°C and 5% CO_2_. For flavivirus detection, the cells were washed with PBS, fixed with 4% PFA, and incubated with anti-E protein Flavivirus group antibody 4G2 in PBS supplemented with 0.3% saponin (Sigma-Aldrich), followed by 30 min incubation with horseradish peroxidase-conjugated goat anti-mouse antibody (Dako) and staining with 3-amino-9-ethylcarbazole substrate (Sigma-Aldrich). For RSV detection, after 72 h, the cells were washed with PBS, fixed with methanol supplemented with 2% H_2_O_2_, and incubated with a biotinylated anti-RSV antibody (Biorad) in PBS containing 1% albumin from bovine serum (Sigma) for 1 h, followed by 30 min incubation with ExtrAvidin Peroxidase (Sigma) and staining with 3,3′-diaminobenzidine substrate (Sigma). Titers were determined by counting plaques and expressed as PFU/ml.

### Paracellular Permeability Assay

The cell layer integrity of fully differentiated cells grown on inserts at the ALI was measured with a permeability assay. Shortly, cells were washed with PBS, and the differentiation medium in the basolateral chamber was replaced with fresh medium. A 2 mg/ml solution of fluorescein isothiocyanate-dextran (FD4, Sigma-Aldrich) was diluted in 150 μl PBS and added to the apical chamber, and the cultures were incubated for 4 h at 37°C and 5% CO_2_. The fluorescence intensity in the basolateral medium was measured with the following wavelengths: excitation 485 nm and emission 544 nm. Fluorescence was expressed relative to an empty 24-well insert used as reference control value.

### Statistical Analysis

Statistical analysis was done using the GraphPad Prism 8 software (GraphPad software, La Jolla, CA, USA). Data are presented as mean and SD, and non-parametric ANOVA (Kruskal-Wallis test) with Dunn’s correction for multiple testing was used to determine differences between groups. A *p* < 0.05 was considered statistically significant.

## Results

### Human NECs Are Susceptible to Infection by Selected Flaviviruses

**Figure 1:**
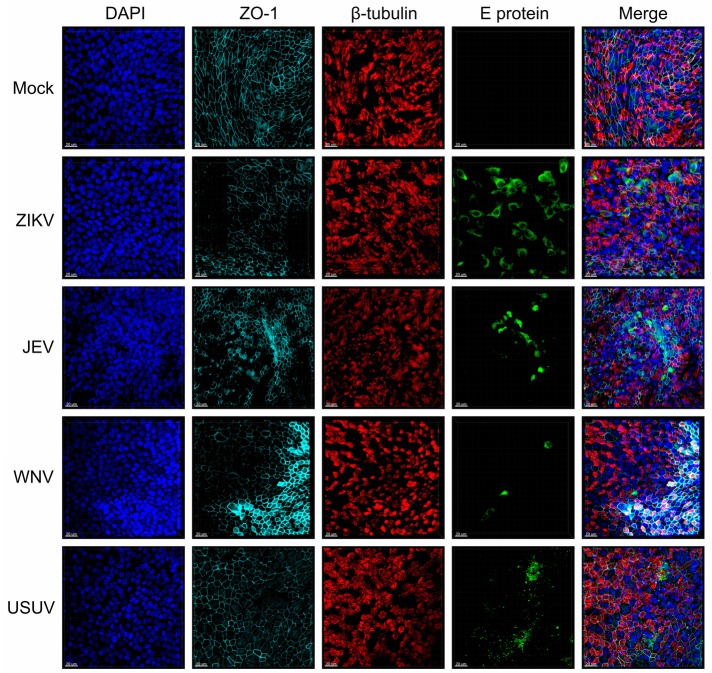
Susceptibility of human NECs to flaviviruses. Confocal microscopy analysis 72 h p.i. with mock, ZIKV, JEV, WNV, and USUV at a MOI of 1 TCID_50_/cell. Blue: DAPI, light blue: ZO-1, red: β-tubulin, green: flavivirus E protein. The micrographs presented are representative of data generated from six independent donors. Bar 20 μm.

To investigate the susceptibility of human NECs toward selected flaviviruses (ZIKV, JEV, WNV, and USUV), we infected NEC cultures from independent healthy donors and visualized flavivirus E protein by confocal microscopy imaging (Figure 1). When compared to mock-infected cells, the infected inserts showed evidence of positive E protein signal 72 h p.i. in ciliated and non-ciliated cells. Of note, depicted areas showing positive virus signal are not representative of the whole insert, as infected cells in nasal ALI cultures were clustered into discrete foci. In addition, some donors did not show any positive staining, indicative of resistance to infection. These results indicate that human NECs cultured at the ALI are susceptible to several emerging flaviviruses.

### Live Flavivirus Particles Are Released Through the Apical and Basolateral Surfaces of NECs

**Figure 2:**
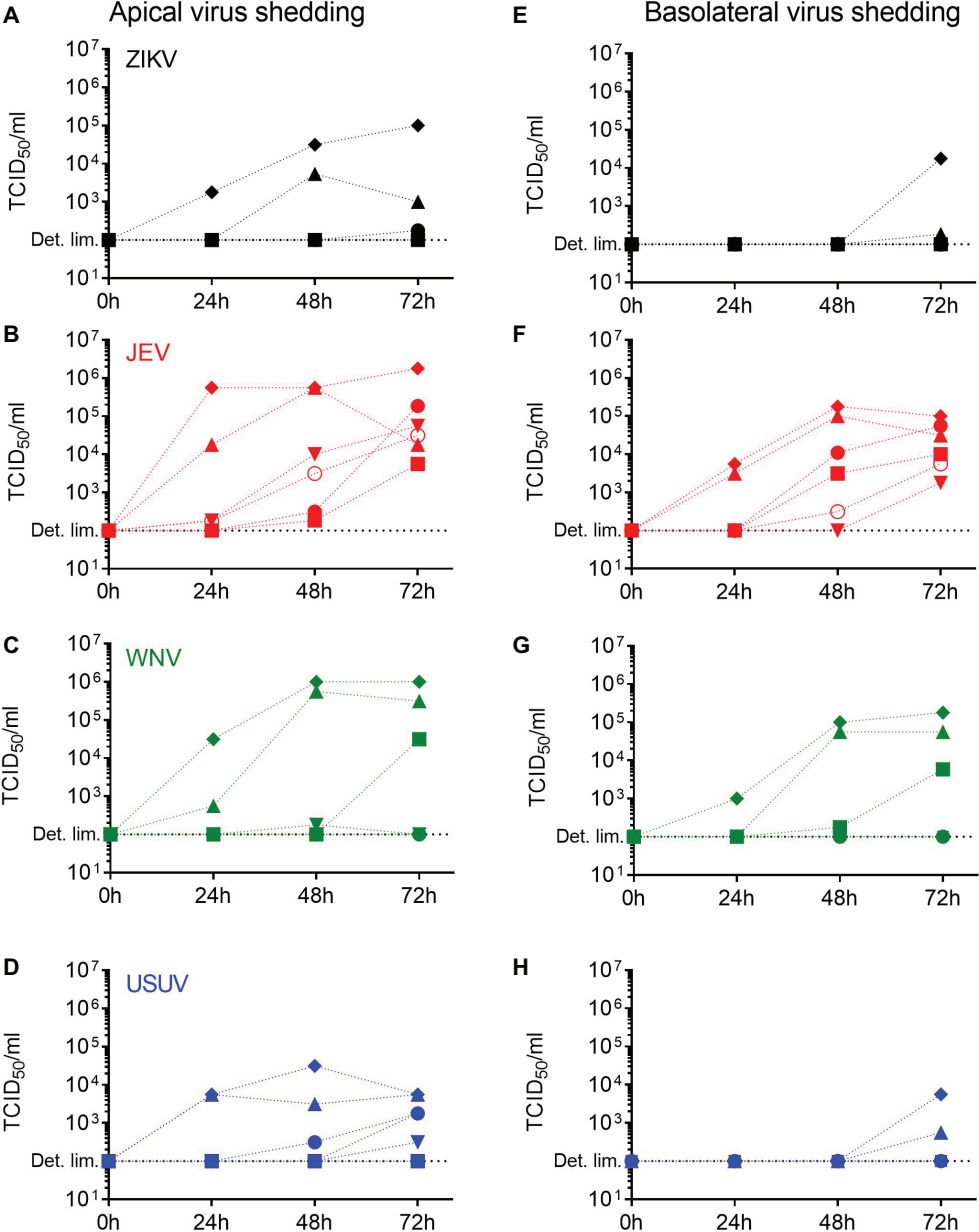
Release of infectious virus by flavivirus-infected human NECs. Apical and basolateral virus shedding up to 72 h p.i. with ZIKV **(A,E)**, JEV **(B,F)**, WNV **(C,G)**, and USUV **(D,H)** measured by TCID_50_ assay in NECs infected at a MOI of 1 TCID_50_/cell. Each symbol represents an independent donor (donor I: dot, donor II: square, donor III: upward triangle, donor IV: diamond, donor V: circle, and donor VI: downward triangle).

Having demonstrated that primary NECs are susceptible to flavivirus infection, we investigated if this was leading to a productive infection. For each selected flavivirus, we measured the levels of live virus released over time in the apical and the basolateral chambers of the NECs using a TCID_50_ assay between 24 and 72 h p.i. We collected apical wash solution and basolateral medium for each donor. In general, the levels of virus released increased over time between 24 and 72 h p.i. and were higher in the apical washings (Figures 2A–D) compared to the basolateral medium (Figures 2E–H) but were dependent on the donor. Importantly, JEV was the only virus for which each donor reached similar levels in both the apical and basolateral media 72 h p.i., meaning the NEC cultures of each donor released JEV infectious particles. Donor IV (diamond) showed the highest levels of shedding for all the viruses. These data indicate that the nasal epithelium has the necessary molecular requirements to allow a full replication cycle leading to the production of infectious flavivirus virions.

### Infection of Human NECs Induces the Transcription of Inflammatory and Antiviral Mediators

**Figure 3:**
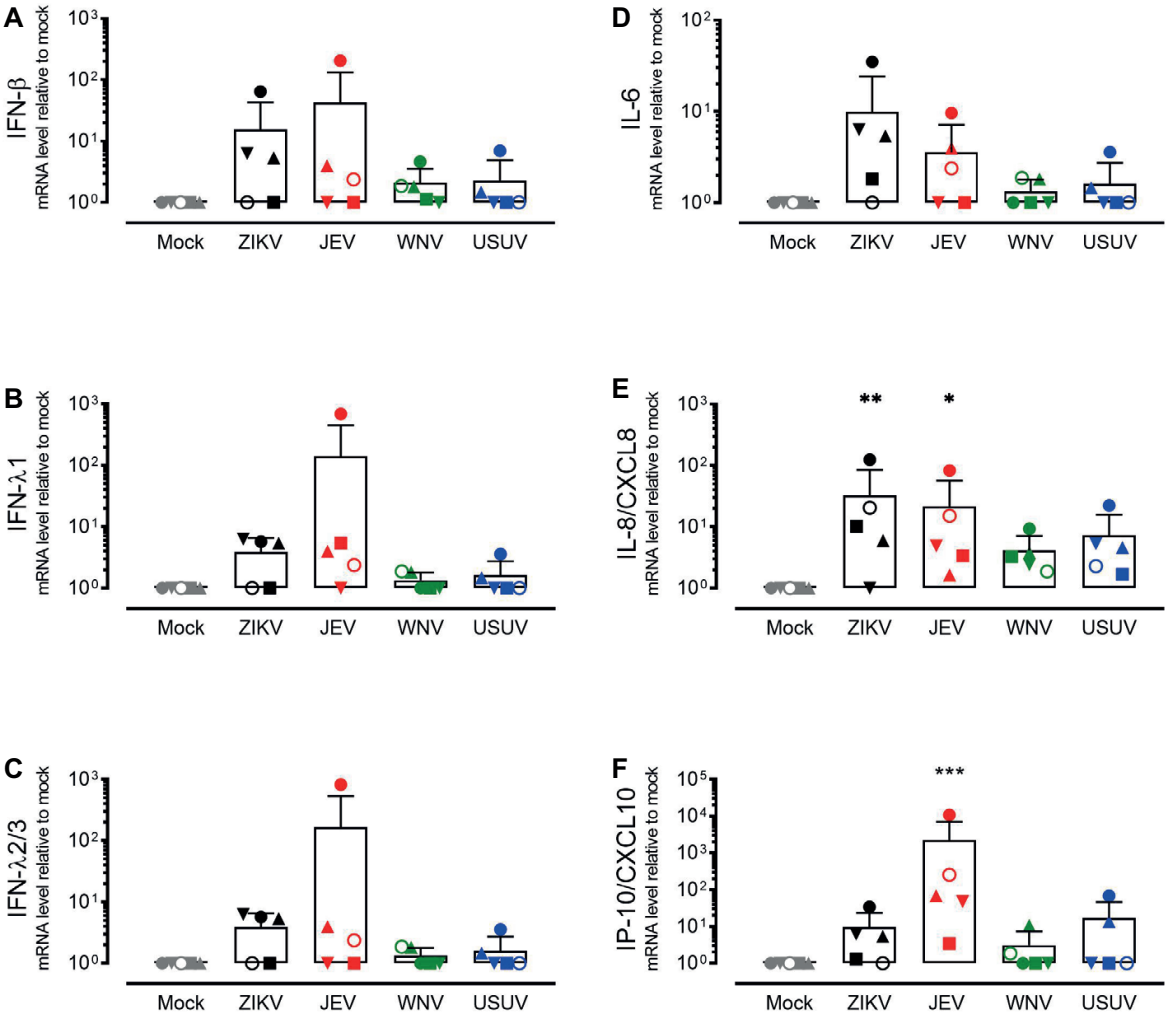
Gene expression levels of antiviral mediators and pro-inflammatory cytokines by NECs after flavivirus infection. IFN-β **(A)** IFN-λ1 **(B)**, IFN-λ2/3 **(C)**, IL-6 **(D)**, IL-8/CXCL8 **(E)**, and IP-10/CXCL10 **(F)** mRNA levels measured by quantitative RT-PCR. Each symbol represents an independent donor (donor I: dot, donor II: square, donor III: upward triangle, donor IV: diamond, donor V: circle, and donor VI: downward triangle). Data are presented as mean and SD and stars indicate significance levels. *p < 0.05; **p < 0.01; ***p < 0.001.

**Figure 4:**
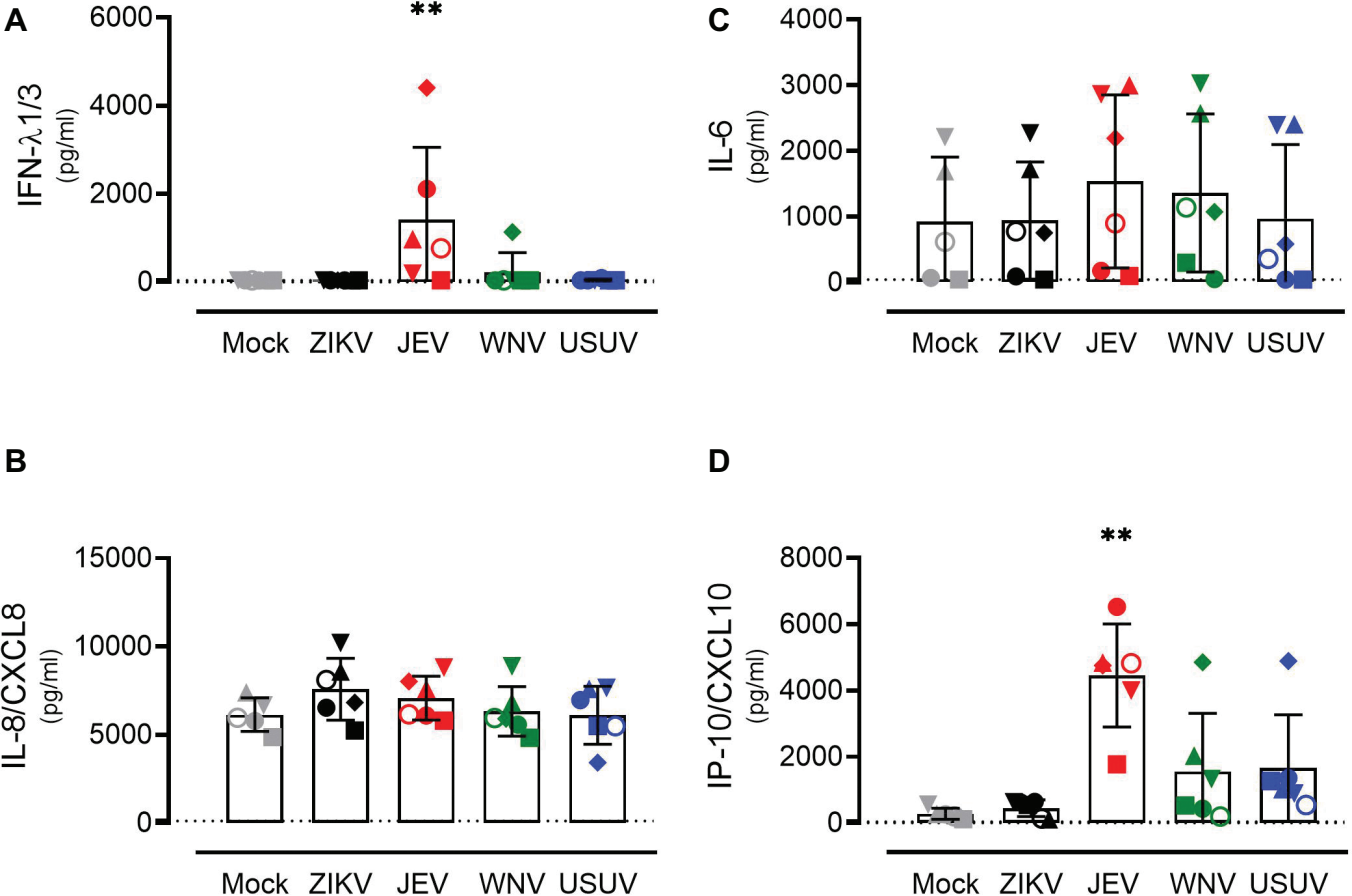
Protein expression levels of antiviral mediators and pro-inflammatory cytokines by NECs after flavivirus infection. IFN-λ1/3 **(A)**, IL-8/CXCL8 **(B)**, IL-6 **(C)**, and IP-10/CXCL10 **(D)** protein levels measured by ELISA. Each symbol represents an independent donor (donor I: dot, donor II: square, donor III: upward triangle, donor IV: diamond, donor V: circle, and donor VI: downward triangle). Data are represented as mean and SD and stars indicate significance levels. **p < 0.01. The dashed lines indicate the detection limits of the ELISA assays (IFN-λ1/3: 40 pg/ml, IL-8/CXCL8: 120 pg/ml, IL-6: 43 pg/ml, and IP-10/CXCL10: 22 pg/ml).

In order to investigate the response of the respiratory epithelium upon flavivirus infection, we measured the expression levels of type I and type III IFNs (Figures 3A–C) and the pro-inflammatory cytokines IL-6, IL-8/CXCL8, and IP-10/CXCL10 (Figures 3D–F). These cytokines were selected based on their upregulation in the course of ZIKV disease in humans as well as their modulation in porcine NECs during JEV infection (Tappe et al., 2016; Garcia-Nicolas et al., 2018). We measured intracellular expression by quantitative RT-PCR 72 h p.i. with ZIKV, JEV, WNV, and USUV and compared it to mock-infected cells. Again, we observed variations in the antiviral response when comparing each donor, but these were less strong than those of live virus release. Donor I (dot) showed the highest expression of type III IFNs, especially after infection with JEV and WNV, although this was not significant (Figures 3B,C). We did not observe a significant increase in IL-6 levels for all flaviviruses in comparison with mock control (Figure 3D). There was a statistically significant increase in the expression of IL-8/CXCL8 after infection with ZIKV and JEV, again with donor I expressing the highest levels (Figure 3E). The expression of IP-10/CXCL10 after JEV infection also reached statistical significance (Figure 3F) when comparing it to mock-infected cells. We observed similar effects of flavivirus infection on cytokine release in the basolateral medium at the protein level that we measured only 72 h p.i. (Figure 4). JEV infection significantly increased protein expression levels of IFN-λ1/3 (Figure 4A) and IP-10/CXCL10 (Figure 4D). There was a trend toward higher release of IL-8/CXCL8 (Figure 4B) and IL-6/CXCL6 (Figure 4C) after JEV infection compared to mock, but this was not significant.

### Epithelial Barrier Function of NECs Is Maintained After Infection With Flaviviruses

**Figure 5:**
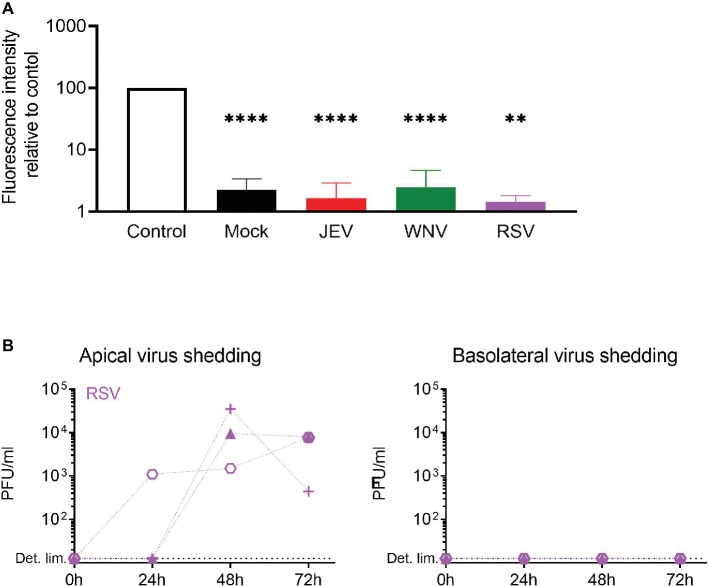
Epithelial barrier function is maintained upon flavivirus infection. **(A)** Relative fluorescence intensity in the basolateral chamber was measured after apical application for 4 h of FD4 on inserts 72 h p.i. with JEV, WNV, and RSV. Values are expressed as % of fluorescence relative to control (empty insert). Data are presented as mean and SD of 3–4 healthy donors. **(B)** Apical and basolateral infectious virus shedding up to 72 h p.i. with RSV measured by PFU assay and expressed in PFU/ml in NECs infected at a MOI of 1 PFU/cell. Each symbol represents an independent donor. Statistical analysis was done in comparison to control; **p < 0.01; ****p < 0.0001.

In order to verify if the infectious virus released in the basolateral chamber of NEC ALI cultures is due to active release or to impaired barrier function, we assessed the tightness of the epithelium upon flavivirus infection with a paracellular permeability assay (Figure 5A). Additionally, we used the respiratory syncytial virus (RSV) – a common pathogen of the upper respiratory tract – since its release upon infection of NEC ALI cultures is limited to the apical side of the epithelium (Villenave et al., 2012). After infecting the ALI cultures of independent donors with JEV, WNV, and RSV and maintaining them in culture for 72 h p.i., we applied fluorescent dextran (FD4) on the cultures and measured the fluorescence intensity in the basolateral chamber. We found that the fluorescence intensity in virus-infected cells was significantly lower compared to control and similar to mock-infected cells. Furthermore, RSV was released from the apical surface of NECs, but we could not detect any infectious RSV in the basolateral medium of NECs, demonstrating the normal function of the epithelium upon infection with a respiratory virus (Figure 5B). Overall, these results indicate that flavivirus infection has no impact on cellular barrier integrity and that infectious virus production through the basolateral surface of NECs is not a consequence of virus-induced leakage of the respiratory epithelium.

## Discussion

Recent case reports of suspected contact transmission raised interest in investigating the oronasal route for flavivirus infection in humans and other vertebrates. Thus, by using a relevant *in vitro* model of the human respiratory epithelium, we evaluated the human upper respiratory tract as a target for flavivirus replication. We performed apical infection of primary human NECs cultured at the ALI with ZIKV, JEV, WNV, and USUV, mimicking oronasal contact. We show that human NECs are susceptible to infection by all the flaviviruses tested and release infectious virus apically and basolaterally in a time- and donor-dependent manner. The replication of flaviviruses in NECs did not damage the epithelial cell layer as demonstrated with a paracellular integrity assay. Additionally, excluding for JEV, flavivirus infection did not induce a significant IFN response in comparison to mock controls and led to the activation of NECs, indicated by an increase in the expression of the inflammatory mediators IL-8/CXCL8 and IP-10/CXCL10.

Until today, human-to-human transmission of ZIKV *via* non-sexual contact has been reported in one case only. It is hypothesized that virus transmission occurred through contact with tears or sweat of the patient who presented a very high viremia (Swaminathan et al., 2016). A similar case of unusual mucocutaneous transmission during medical care was also reported for DENV (Chen and Wilson, 2016). In addition, non-vector borne transmission of WNV is suspected among turkey breeder farm workers (Centers for Disease Control and Prevention, 2003). Contact transmission could play a role in densely populated areas in individuals at risk such as pregnant women and health care personnel and where human come into close contact with animal species. It is important to mention that DENV and JEV RNAs have only recently been detected in the upper respiratory track of human patients (Cheng et al., 2017; Bharucha et al., 2018). While of limited physiological relevance, previous investigations indicated that the human respiratory cell lines Hep-2, Calu-3, and A549 are susceptible to ZIKV infection (Chan et al., 2016; Frumence et al., 2016). Nevertheless, the risk of direct contact transmission in humans is unclear, and there is a lack of *in vivo* evidences. In non-human vertebrates, direct transmission of JEV and ZIKV was reported in pigs and guinea pigs, respectively, and JEV RNA was also detected in oral fluid samples of pigs and miniature swines (Ricklin et al., 2016a; Deng et al., 2017; Lyons et al., 2018). Also, non-vector borne transmission has been described for other flaviviruses by close contact between animals, including WNV infection in geese (Austin et al., 2004), Bagaza virus in partridges (Llorente et al., 2015), or Tembusu virus in ducks (Li et al., 2015). In our setting, infectious viral particles of all the flaviviruses tested were shed by infected human NECs in both the apical and the basolateral compartments, with particularly high levels for JEV and to a lesser extent for WNV. By checking the paracellular permeability of the epithelial layer upon infection, we could show that virus is actively shed and not released due to a leaky epithelium. This suggests the potential of virus particles to initiate a systemic infection after replicating in the upper respiratory tract epithelium in humans. In this regard, the mucosal surface of the respiratory tract could act as a target for flaviviruses to further spread to other susceptible cell types and tissues. Along this line, assuming the high levels of infectious flavivirus shedding by infected human NECs, we were surprised to measure modest IFNs and pro-inflammatory cytokine responses and no evidence of epithelial injury, pointing on flavivirus evasion mechanisms. Replacing this observation in its physiological context, an inappropriate signalization by the infected upper respiratory epithelium to the surrounding immune microenvironment could promote the spread of infection to other cellular compartments. In order to confirm our *in vitro* findings, it would be of great interest to screen nasopharyngeal swabs from flavivirus-infected individuals during the first stages of infection as suggested by DENV and JEV RNA detection in upper respiratory tract in human cases (Cheng et al., 2017; Bharucha et al., 2018). Our investigations also indicate that this model could be used to evaluate the potential of other zoonotic viruses like Rift Valley fever (genus *Phlebovirus*) and Venezuelan equine encephalitis (genus *Alphavirus*) viruses to be transmitted to individuals in close contact with cattle and horses.

Although a great effort is put in trying to understand the biology of flaviviruses, the exact molecular mechanisms of cell entry remain largely unknown. Common receptors include the TAM receptor family members, AXL or the C-type lectin DC-sign (Perera-Lecoin et al., 2013). Upon virus entry, infected cells are activated by virus-associated products and trigger the expression of antiviral mediators including IFNs and inflammatory cytokines. It is known that flaviviruses antagonize the IFN signaling pathway *via* several mechanisms including non-structural proteins or non-coding viral RNAs, small flavivirus RNAs (sfRNAs), highly conserved among flaviviruses (Miorin et al., 2017). In our model of flavivirus infection of the human respiratory epithelium, we noticed differences in susceptibility of airway epithelial cells obtained from different donors. This demonstration of resistance to infection can be attributed to host factors that are variably effective in each donor to interfere with virus infection and/or replication. Among these, genetic and metabolic factors have been proposed in the context of *in vitro* infection with influenza (Travanty et al., 2015). When looking at flavivirus infection, there is experimental evidence of variations in susceptibility of dendritic cells from different donors, which may reflect the differences in flavivirus pathogenesis in human (Bowen et al., 2017; Vielle et al., 2018). Following these concepts, there was no significant expression of type I and type III IFNs in comparison to mock control upon infection of human NEC cultures with most of the flaviviruses tested. In contrast, a significant upregulation of IL-8/CXCL8 and IP-10/CXCL10 was detected upon infection with JEV and ZIKV. Of interest, these cytokines were detected in the serum of humans during the acute and/or recovery phases of ZIKV infection and are involved in the recruitment of immune cells by porcine NECs during JEV infection leading to the amplification of virus replication (Tappe et al., 2016; Garcia-Nicolas et al., 2018).

In summary, this is the first report providing information on the susceptibility of the human upper respiratory epithelium to flavivirus infection using a physiologically relevant *in vitro* system. Thus, we propose respiratory epithelial cells of the nasal cavity as cellular targets for flavivirus replication. Consequently, it is conceivable that the replication of flaviviruses in the human upper respiratory epithelium could potentially lead to the spread of infection to other body compartments through basolateral virus release. Our data suggest that the interaction of flaviviruses at mucosal surfaces should be further investigated to evaluate the risks of alternative routes of flavivirus transmission in human. Finally, our data are of relevance for safety precautions to protect individuals potentially exposed to flaviviruses such as laboratory personnel, healthcare providers, and animal caretakers.

## Data Availability

The datasets generated for this study are available on request to the corresponding author.

## Author Contributions

MA and OG-N conceptualized the study. NV, OG-N, BO and MB performed the investigation of the study. NV and OG-N wrote the original draft. MA administrated the project and contributed to funding acquisition with AS. All authors reviewed and edited the manuscript.

### Conflict of Interest Statement

The authors declare that the research was conducted in the absence of any commercial or financial relationships that could be construed as a potential conflict of interest.
